# Yinhua Pinggan Granules alleviate lung and intestinal damage in influenza virus-infected mice by modulating gut microbiota and its metabolites to activate the GPR43-MAVS-IRF3-IFN-β pathway

**DOI:** 10.3389/fmicb.2025.1532108

**Published:** 2025-10-06

**Authors:** Di Zeng, Huifen Zhou, Haitong Wan, Jing Chen

**Affiliations:** ^1^School of Life Science, Zhejiang Chinese Medical University, Hangzhou, Zhejiang, China; ^2^Zhejiang Key Laboratory of Chinese Medicine for Cardiovascular and Cerebrovascular Disease, Zhejiang Chinese Medical University, Hangzhou, Zhejiang, China; ^3^School of Basic Medical Sciences, Zhejiang Chinese Medical University, Hangzhou, Zhejiang, China; ^4^School of Chinese Medical Sciences, Henan University of Chinese Medicine, Zhengzhou, Henan, China

**Keywords:** Yinhua Pinggan Granules, H1N1, intestinal flora, short-chain fatty acids, GPR43, type I interferon

## Abstract

**Objective:**

Derived from Ma Huang Decoction in the *Shang Han Lun*, Yinhua Pinggan Granules (YHPG) are used in traditional Chinese medicine for treating influenza. This study highlights the gut microbiota’s role in intestinal damage and acute lung injury from influenza virus infection, offering insights into influenza A virus prevention and treatment through the gut-lung axis.

**Methods:**

Using a mouse model disrupted by a four-antibiotic regimen, we assessed survival, weight, lung, and spleen indices post-IAV infection. We evaluated lung and intestinal pathology, viral load, and protein expressions via H&E staining, RT-qPCR, and immunofluorescence. 16S rRNA sequencing and targeted metabolomics were utilized to uncover the impact of YHPG treatment on disrupted gut microbiota and its metabolites after H1N1 infection.

**Results:**

H&E staining showed severe lung and intestinal damage in IAV-infected mice with disrupted gut microbiota. Immunofluorescence results demonstrated that relative depletion of gut microbiota might exacerbate colonic barrier damage in IAV-infected mice. YHPG restored microbiota diversity, increasing SCFA-producing bacteria, aligning with metabolite changes. Western blot and RT-qPCR showed activation of the GPR43-MAVS-IRF3-IFN-I pathway, linked to SCFA regulation.

**Conclusion:**

YHPG alleviate influenza symptoms, promoting SCFA-producing bacteria and maintaining gut homeostasis. They modulate the GPR43-MAVS-IRF3-IFN-β pathway, suggesting novel treatment avenues for influenza through gut microbiota modulation.

## 1 Introduction

Respiratory virus infections, such as COVID-19, present serious challenges to human health, with influenza A virus epidemics and pandemics also posing a significant global threat (Javanian et al., 2021). Influenza, an acute viral respiratory infection, is marked by high morbidity and mortality rates. The influenza virus, part of the Orthomyxoviridae family, primarily comprises the surface glycoprotein hemagglutinin (HA) and the envelope protein neuraminidase (NA) (Zhu et al., 2023). The virus enters the host through respiratory droplets or contact with oral or nasal mucosa. Sialic acid receptors capable of binding the virus are widely distributed on various cell surfaces in lung tissue, the respiratory tract, including epithelial cells, lymphocytes, monocytes. The HA binds to these sialic acid receptors on the host cell surface, promoting endocytosis and facilitating viral entry into the host cell (te Velthuis and Fodor, 2016; Ni et al., 2024). Subsequently, vRNPs and other proteins are synthesized and assembled into progeny virus particles, which are then released and spread in the lungs (Creager et al., 2018).

Following infection with the influenza virus, replication primarily occurs in pulmonary epithelial cells, stimulating the innate immune system to produce pro-inflammatory cytokines. An uncontrolled inflammatory response may lead to a cytokine storm and multi-organ damage, with the level of the cytokine storm determining the severity of disease during influenza infection (Gu et al., 2019). Concurrently, various pattern recognition receptors (PRRs) in innate immune cells—such as macrophages—activate the phosphorylation of interferon regulatory factors 3 and 7 (IRF3, IRF7). This initiates type I interferon (IFN-I) responses, which are a critical component of the innate immune reaction. For instance, RIG-I-MAVS detects the 5′-triphosphorylated viral ssRNA produced post-replication; RIG-I then binds ATP, interacting with mitochondrial antiviral signaling protein (MAVS) (Iwasaki and Pillai, 2014). Influenza infection can also activate the NLRP3 inflammasome, composed of NLRP (or other PRRs), the adaptor ASC, and pro-caspase 1 (Swanson et al., 2019). MAVS can promote the recruitment of NLRP3 to mitochondria, where ATP facilitates the interaction between MAVS and NLRP3, specifically promoting the inflammasome-dependent production of IL-1β. After viral infection, MAVS protein can induce the formation of high molecular weight aggregates, which are effective activators of IRF3 (Subramanian et al., 2013).

Recent studies have indicated that the antiviral response of IFN-I can be attributed to the gut microbiota. The severity of influenza infection is closely linked to the heterogeneous responses of the gut microbiota. In the experiments of influenza infection and fecal microbiota transplantation, the abundance of *Bifidobacterium pseudomolium* and *Bifidobacterium animalis* in the intestinal microbiota significantly increased, thereby enhancing the host’s resistance to influenza. *Bifidobacterium* has the potential to serve as a novel biomarker for predicting influenza severity and patient mortality (Zhang et al., 2020). The gut microbiota can also induce colonic dendritic cells to produce IFN-I through the TLR4-TRIF signaling pathway, thereby regulating and enhancing the immune response against pathogens (Stefan et al., 2020). Moreover, the microbial metabolite desaminotyrosine (DAT) produced by clostridia can further strengthen antiviral responses by amplifying IFN-I signaling (Steed et al., 2017). Short-chain fatty acids (SCFAs), as metabolites of the gut microbiota and endogenous agonists in GPCR signaling pathways, may help prevent pulmonary viral infections and maintain intestinal homeostasis and barrier integrity, making them potential new therapeutic targets for viral infections (Smith et al., 2013; Feng et al., 2023). Acetate can prevent RSV-induced diseases by enhancing type I interferon responses and increasing the expression of interferon-stimulated genes (ISGs) in pulmonary epithelial cells through the activation of membrane receptor GPR43 (Antunes et al., 2019). Acetate and propionate derived from Blautia can induce IFN-I responses in macrophages at mucosal and extraintestinal sites, thereby providing protection against intestinal viral infections (Wang G. et al., 2023; Wang L. et al., 2023).

Yinhua Pinggan Granules (YHPG) is a patented formula of traditional Chinese medicine approved by the National Medical Products Administration (Registration No. Z20133007) for the treatment of colds, viral pneumonia and related diseases (Patent No. ZL03151188.0), and it has also obtained the Traditional Chinese Medicine New Drug Certificate (State Drug License No. Z20120004). YHPG is developed from modifications of the “Mahuang Decoction” found in the classical Chinese text “*Treatise on Febrile Diseases*,” consisting of six herbal ingredients: *Lonicera japonica Thunb.*, *Reynoutria japonica Houtt.*, *Pueraria alopecuroides Craib*, *Prunus armeniaca L.*, *Ephedra intermedia Schrenk & C. A. Mey.* and *Glycyrrhiza uralensis Fisch. ex DC.*, in a ratio of 4: 4: 4: 2: 2: 1.

Preliminary studies conducted by the research team have utilized high-resolution liquid chromatography coupled with mass spectrometry (HPLC-Q-Exactive MS) to perform comprehensive qualitative analysis, to determine the contents of active components, and assess the pharmacokinetics, thereby providing a solid foundation for its clinical application (Yalkun et al., 2024). It was found that the main active components of YHPG were chlorogenic acid, puerarin, polydatin, 3′-methoxypuerarin, emodin and glycyrrhizic acid. Among them, chlorogenic acid, which has the highest content, has antiviral and antioxidant properties, promotes the growth of probiotics and fights against intestinal flora imbalance (Singh et al., 2023). Puerarin can be used as an NA blocker to inhibit influenza A virus (Wang et al., 2021). Polydatin has been shown to have inhibitory effects on neuraminidase, as well as anti-inflammatory and anti-apoptotic pharmacological effects (Karami et al., 2022; Wang G. et al., 2023; Wang L. et al., 2023). Emodin has a significant anti-influenza A virus effect, which can significantly inhibit the replication of H1N1, reduce the expression of TLR2/3/4/7, MyD88 and TRAF6 induced by viral infection, and reduce the phosphorylation of p38/JNK MAPK and nuclear translocation of NF-κB p65 (Dai et al., 2017). We also conducted a network pharmacological study on emodin and found that emodin has potential pharmacological effects in the treatment of COVID-19 infection (Du et al., 2021). Current studies indicate that Glycyrrhizic acid can fight a wide range of viruses, and its mechanism may be related to the inhibition of the viral replication by interfering with protein transcription factors such as NF-κB, p38 and JNK (Langer et al., 2023).

In the clinical trial, we employed randomized, double-blind, active-controlled and multicenter design. The study enrolled 416 patients with influenza complicated by upper respiratory tract infection. Results demonstrated that YHPG significantly alleviated symptoms including fever, headache, myalgia, nasal congestion, rhinorrhea, cough, and sputum production (He et al., 2019). In both *in vivo* and *in vitro* studies, YHPG has been demonstrated to protect against IAV infection in mice by modulating the gut microbiota and enhancing short-chain fatty acid (SCFA) production (Yang et al., 2024). Its antiviral mechanism is further supported by its ability to inhibit the TLR4-MyD88-TRAF6 signaling pathway, suppress NF-κB p65 activation, and regulate apoptosis-related factors (Peng et al., 2016a, 2016b; Du et al., 2020).

In this study, we aimed to investigate the relationship between YHPG treatment of IAV-infected mice with lung and intestinal injuries, gut microbiota, and their derived metabolites, short-chain fatty acids (SCFAs). The research found that YHPG could alleviate acute lung injuries and intestinal barrier damage caused by viral infections, regulate the composition and abundance of gut microbiota in mice, increase the relative abundance of SCFA-producing bacteria, and enhance SCFA levels in the body. Further confirmation of YHPG’s regulation of the GPR43-MAVS-IRF3-IFN-β signaling pathway was obtained through Western blotting and RT-qPCR. Acetate and propionate may interact with GPR43 and activate NLRP3, leading to the oligomerization of MAVS, phosphorylation of TBK1 and IRF3, and simultaneous induction of IFN-I, thereby enhancing the host’s antiviral capacity. This may provide a reference for designing intervention strategies against respiratory viral infections.

## 2 Materials and methods

### 2.1 Materials and reagents

YHPG Granules were formulated using *Lonicera japonica Thunb*. (10 g), *Reynoutria japonica Houtt*. (10 g), *Pueraria alopecuroides Craib* (10 g), *Prunus armeniaca L*. (5 g), *Ephedra intermedia Schrenk & C. A. Mey*. (5 g), and *Glycyrrhiza uralensis Fisch. ex DC* (2.5 g). The drug (Batch No. 200404) is provided by Shaanxi Dongke Pharmaceutical Co., Ltd. (Xiangyang, China), dissolved in distilled water to make its crude drug concentration 0.85 g / mL and sterilized with a 0.22 μm filter. Oseltamivir phosphate (M1301) was obtained from Basel Hofmann-La Roche.

### 2.2 Virus

The mouse-adapted H1N1 strain A/Puerto Rico/8/1934 was kindly provided by the Hangzhou Institute of Medicine, Chinese Academy of Sciences. Viral titers were determined using the TCID50 method. Briefly, cells were infected with serial dilutions of the viral suspension, and cytopathic effects were observed. The TCID50 value was calculated according to the Reed-Muench method, and the result was 10^3.4^.

### 2.3 Mice

Male ICR mice (18 ± 2 g, pathogen-free) were obtained from Zhejiang Provincial Experimental Animal Center (License No. SYXK (Zhe) 2023-0005). Mice were housed in an SPF environment (20–22 °C, 45% ± 5% humidity, 12-h light–dark cycle) with food and water provided ad libitum. All experiments were approved by the Animal Ethics Committee of the Hangzhou Institute of Medicine, Chinese Academy of Sciences (Ethics No. 2023R0051). All mice were acclimatized for 1 week before modeling.

### 2.4 Virus infection of mice and groups

Mice were anesthetized with isoflurane and infected intranasally with 10 × LD50 of H1N1 virus in 50 μL PBS. Mice were monitored daily for weight loss. They were divided into Control, H1N1, Oseltamivir, and YHPG groups (n = 6 per group), with medication administered once daily for 5 days post-infection. Control and H1N1 groups received saline gavage.

### 2.5 Depletion of commensal bacteria and groups

As previously described, to deplete the intestinal microbiota, mice were treated via gavage with vancomycin (100 mg/kg), neomycin sulfate (200 mg/kg), metronidazole (200 mg/kg), and ampicillin (200 mg/kg) at a frequency of twice daily for seven consecutive days to establish the ABX mouse model. Mouse feces (~0.05 g/mouse) were collected in 1 mL of sterile PBS and homogenized; after brief centrifugation, the supernatant was inoculated onto GAM plates and cultured anaerobically at 37 °C in an incubator (80% N_2_: 10% CO_2_: 10% H_2_) for 48 h. Bacterial counting was performed using the colony-forming unit method, achieving a removal efficiency of >99.9%. Additionally, 16S sequencing was conducted to analyze the intestinal microbiota. All antibiotics used in this study were purchased from Shanghai Macklin Biochemical Co., Ltd. and Shanghai Aladdin Biochemical Technology Co., Ltd.

ABX mice were randomly assigned into four groups: ABX-Control group, ABX-H1N1 group, ABX-Oseltamivir group, and ABX-YHPG group. The infection and administration methods were consistent with the previous protocol. After 5 days of treatment, the mice were euthanized, and blood, colon tissue, lung tissue, cecal contents, and feces were collected for further analysis.

### 2.6 Histopathological examination

Colon and lung tissues were fixed in 4% paraformaldehyde, dehydrated, and embedded in paraffin. Thin sections (4 μm) were sliced from the paraffin blocks using a microtome and mounted on glass slides. The sections were stained with hematoxylin and eosin (H&E), and pathological changes in the colon and lung tissues were observed under a light microscope. Images were captured using an optical microscope (Hamamatsu, Japan), and analysis was performed using NDP. View2 software.

### 2.7 Enzyme-linked immunosorbent assay

Peripheral blood was collected through puncture of the fundus venous plexus in mice. After centrifugation at 3,000 rpm for 10 min at 4 °C, the serum was isolated for enzyme-linked immunosorbent assay analysis. Protein levels of IL-10, IL-1β, IL-6, TNF-α, and IFN-β were analyzed according to the manufacturer’s instructions. ELISA kits for mouse IL-10, IL-1β, IL-6, TNF-α, and IFN-β were purchased from Enzyme-linked Biotechnology Co., Ltd. (Jiangsu, China).

### 2.8 Immunofluorescence experiment

To assess and visualize occludin expression in colon tissues, immunofluorescence staining was performed on paraffin-embedded sections. The sections were deparaffinized, rehydrated, and blocked with bovine serum albumin to prevent nonspecific antigen binding. Primary antibody against occludin (ab216327, Abcam) was diluted and applied, with overnight incubation at 4 °C in a humid chamber. The sections were washed three times with PBS (5 min each), followed by incubation with a FITC-conjugated goat anti-mouse IgG (H&L) secondary antibody (GB22301, Servicebio) at a 1:300 dilution for 50 min in the dark at room temperature. The nuclei were counterstained with DAPI, and tissue autofluorescence was quenched. Fluorescent images were captured using a fluorescence scanning system (VS120-S6-W, OLYMPUS) and processed with OlyVIA 3.3 software. Three microscopic fields from each tissue section were analyzed, and the percentage of positive staining was quantified using Image J software (National Institutes of Health, United States).

### 2.9 Quantitative real-time PCR

RNA from lung and colon tissues was extracted using TRIzol (12183-555, Thermo Fisher) and then reverse transcribed using the SuperScript™III First-Strand Synthesis SuperMix kit (11752-050, Thermo Fisher). cDNA was amplified using the Power SYBR® Green PCR Master Mix (4367659, Applied Biosystems) reaction system and quantified using a fluorescence quantitative PCR instrument (CFX384, Bio-Rad). The results were normalized to the housekeeping gene β-actin (ΔCt). The relative levels of the target genes in the control group were plotted as 1.0 using the 2^−ΔΔCt^ method. In HA, NP, M1 detection, ABX-H1N1 was normalized. The primer sequences are shown in Table 1.

**Table 1:** The primers for RT-qPCR.

Genes	Forward primer (5′-3′)	Reverse primer (5′-3′)
M1	GACTCCCAGCATCGGTCTCA	CTCTGCTGCTTGCTCACTCGAT
HA	GCTTCCAGTGAGATCATGGTCCTA	GCTCCCTCAGCTCCTCATAG
NP	GGGTCGGTTGCTCACAAGTC	CCGACTAGAGAGTATCCCTCTCTT
β-actin	GTGTGACGTTGACATCCGTAAAGA	GCCGGACTCATCGTACTCCT
ZO-1	CCATGACTCCTGACGGTTGGTCTT	CGGATCTCCAGGAAGACACTTGT
Occludin	GCGATCATACCCAGAGTCTTTC	GGTGTCTCTAGGTTACCATTGC
Claudin-1	CCTGCCCCAGTGGAAGATTTACT	GTGCTTTGCGAAACGCAGGACAT
GPR43	GGTGTTCAGTTCCCTCAATGC	GCATAGAGGAGGCAGGATTG
NLRP3	GCAGGCATCGGGAAAACC	CTCTCGGCAGTGGATAAAGAACAAA
MAVS	CTGCCAACACAATACCACCTGAG	CTCTGGTCCAGAGTGCAAGCT
TBK1	GACATGCCTCTCTCCTGTAGTC	GGTGAAGCACATCACTGGTCTC
IRF3	CGGAAAGAAGTGTTGCGGTTAGC	CAGGCTGCTTTTGCCATTGGTG

### 2.10 Bacterial profiling by sequencing analysis of 16S rRNA

After extracting total DNA from cecal fecal samples and quality control, the V3-V4 region of bacterial 16S rRNA was amplified using primers 338F (5′-ACTCCTACGGGAGGCAGCA-3′) and 806R (5′-GGACTACHVGGGTWTCTAAT-3′). The DNA products were confirmed through 1.2% agarose gel electrophoresis, purified, and amplified. Paired-end sequencing of community DNA was performed on the Illumina NovaSeq platform. After removing primer sequences and unmatched primer fragments, quality control, denoising, merging, and chimera removal were performed using QIIME2. Once denoising of all libraries was completed, ASV feature sequences and ASV tables were merged, and singleton ASVs were removed. Sequence length distribution statistics were conducted using R, and the length distribution of high-quality sequences contained in all samples was ultimately analyzed.

### 2.11 Quantification of SCFAs by GC/MS

An appropriate amount of fecal sample was placed in a 1.5 mL centrifuge tube, to which 500 μL of water and 100 mg of glass beads were added. The mixture was homogenized for 1 min and then centrifuged at 12,000 rpm for 10 min at 4 °C. From the supernatant, 200 μL was taken and mixed with 100 μL of 15% phosphoric acid, followed by the addition of 20 μL of an internal standard solution (4-methylvaleric acid) at 375 μg/mL and 280 μL of ether, homogenized for 1 min, and then centrifuged again at 12,000 rpm for 10 min at 4 °C. The supernatant was subjected to testing. Chromatographic conditions included a Thermo Trace 1300 gas chromatography system (Thermo Fisher Scientific, United States) with an Agilent HP-INNOWAX capillary column (30 m × 0.25 mm ID × 0.25 μm). A split injection was performed with an injection volume of 1 μL and a split ratio of 10:1. The injector temperature was set at 250 °C, the ion source temperature at 300 °C, and the transfer line temperature at 250 °C. The program started at 90 °C, then increased to 120 °C at a rate of 10 °C/min, followed by a rise to 150 °C at 5 °C/min, and finally increased to 250 °C at 25 °C/min, holding for 2 min. The carrier gas was helium with a flow rate of 1.0 mL/min. Mass spectrometry conditions were set with the Thermo ISQ 7000 mass spectrometer (Thermo Fisher Scientific, United States) using an electron impact ionization (EI) source in SIM scanning mode with an electron energy of 70 eV. The standards used for analysis, including acetic acid, propionic acid, butyric acid, isobutyric acid, valeric acid, isovaleriac acid, hexanoic acid, and isohexanoic acid, were purchased from Sigma-Aldrich (Missouri, United States).

### 2.12 Western blot

Total protein was extracted using a total protein extraction kit (78510, Thermo Pierce), and protein concentrations were determined using a BCA protein assay kit (P0010, Biyuntian). Proteins were separated by sodium dodecyl sulfate-polyacrylamide gel electrophoresis (SDS-PAGE) and transferred to polyvinylidene fluoride (PVDF) membranes. Membranes were blocked with 5% BSA in TBST for 1 h at room temperature, followed by incubation overnight at 4 °C with primary antibodies. Membranes were then washed with TBST four times and incubated with secondary antibodies at room temperature for 1 h, followed by five washes with TBST. Detection was performed using SuperSignal® West Dura Extended Duration Substrate (34075, Thermo Pierce), and membranes were exposed to X-ray film for 5–10 min before developing and fixing. Band optical density values were analyzed using Image J software, with three replicates per band. Relative protein expression was calculated as (target protein optical density value / internal reference optical density value) × 10n, with results presented as mean ± standard deviation. The following antibodies were used: GPR43 (19952-1-AP), TBK1 (28397-1-AP) from Proteintech (Chicago, United States); NLRP3 (ab181602), MAVS (ab189109), β-actin (ab8226) from Abcam (Cambridge, UK); and p-TBK1 (5483), p-IRF3 (29047), IRF3 (4302) from CST.

### 2.13 Quantification and statistical analysis

Data were analyzed using GraphPad Prism v9.5 software (La Jolla, CA, United States). Results are presented as mean ± standard deviation (SD). One-way ANOVA with Tukey’s *post-hoc* test or Kruskal-Wallis test or Dunnett’s *post-hoc* test was used for three or more groups. Survival curves were compared using the Kaplan–Meier method, and *p* < 0.05 was considered statistically significant.

## 3 Results

### 3.1 YHPG Granules alleviate infection symptoms and protect lung and intestinal tissues in IAV-infected mice

**Figure 1:**
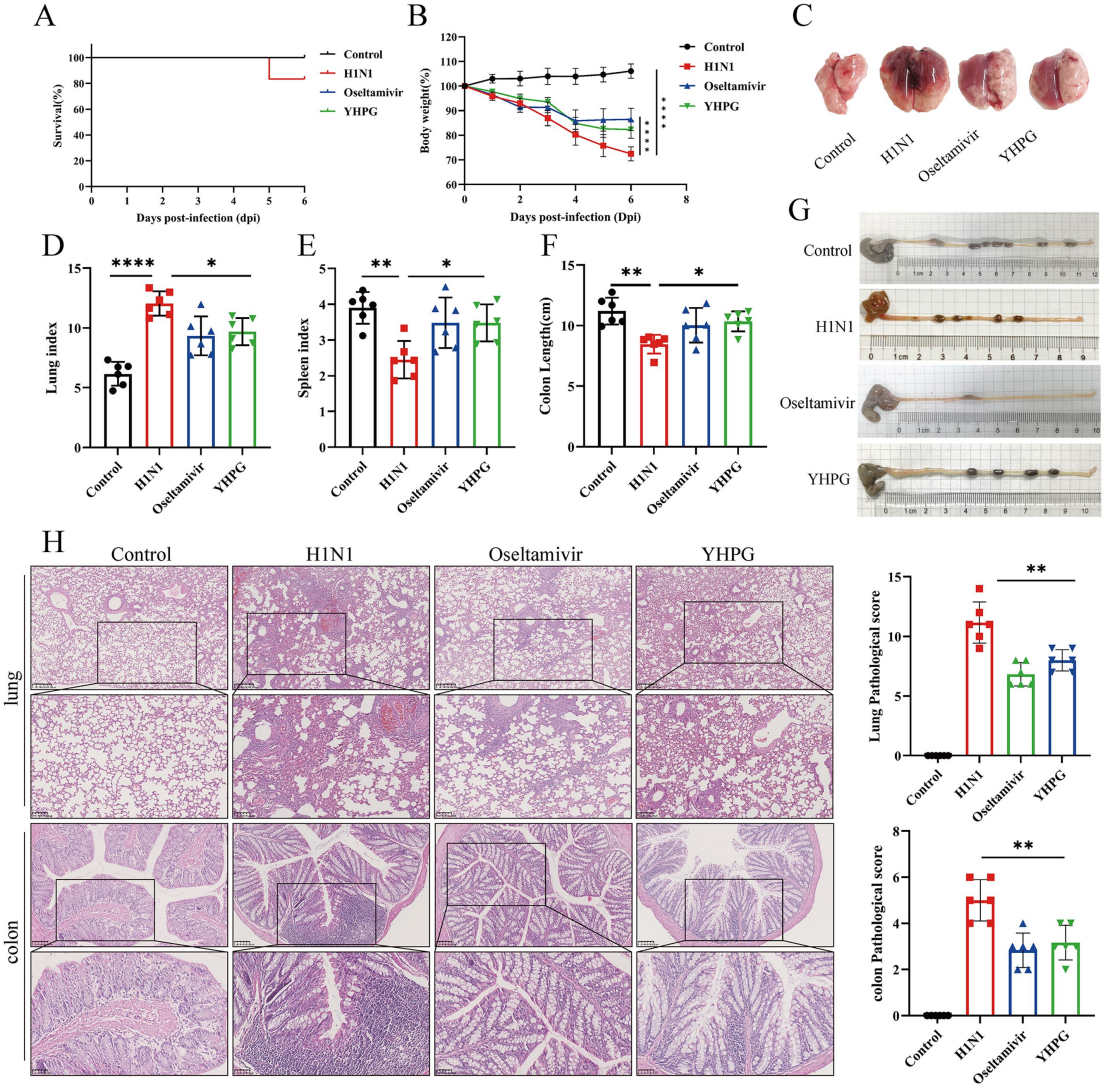
Effects of YHPG treatment on IAV-infected mice. **(A)** Survival rates of animals after infection. **(B)** Relative changes in body weight of mice during the experimental period. **(C)** Representative images of the overall morphology of mouse lung tissue (*n* = 6). **(D)** Lung index = Lung Weight (mg) / Body Weight (g). **(E)** Spleen index = Spleen Weight (mg) /Body Weight (g). **(F)** Statistics on colon length in mice. **(G)** Representative images of mouse colon length (*n* = 6). **(H)** Representative images of H&E staining in lung and intestine samples and the Pathological score (scale bars: 250 μm, 100 μm, 50 μm). *p < 0.05, **p < 0.01, ***p < 0.001, ****p < 0.0001.

To evaluate the therapeutic effect of YHPG Granules on IAV-infected mice, 18 healthy ICR mice were intranasally infected with H1N1 influenza virus and randomly divided into three groups: H1N1, oseltamivir phosphate, and YHPG. An uninfected control group was also established. Based on previous studies, the optimal dosage of YHPG was determined to be 11 g/kg, and this dosage was administered to the YHPG group for 5 days post-infection. Compared to the Control group, the H1N1 group showed mortality by day 5 post-infection (Figure 1A), with significant weight loss, increased lung index, decreased spleen index, and evident pulmonary edema and congestion (Figures 1B–E; Supplementary Figure S3), along with a significant shortening of the colon (Figures 1F,G). Both YHPG and oseltamivir treatment alleviated these symptoms to varying degrees, reducing pulmonary congestion, edema, and colon shortening caused by the viral infection. Histological analysis showed no structural changes in the lungs of the control group, while the H1N1 group exhibited marked lymphocytic infiltration around small blood vessels and bronchioles, with widened alveolar septa. YHPG and Oseltamivir treatments significantly reduced the inflammatory infiltration areas. H&E staining of the colon revealed intact and compactly arranged intestinal villous epithelial cells in the control group. In contrast, the H1N1 group displayed significant neutrophil and lymphocyte infiltration, while both YHPG and oseltamivir treatments significantly reduced lymphocytic infiltration (Figure 1H).

### 3.2 YHPG Granules alleviate infection symptoms and protect lung and intestinal tissues in ABX IAV-infected mice

**Figure 2:**
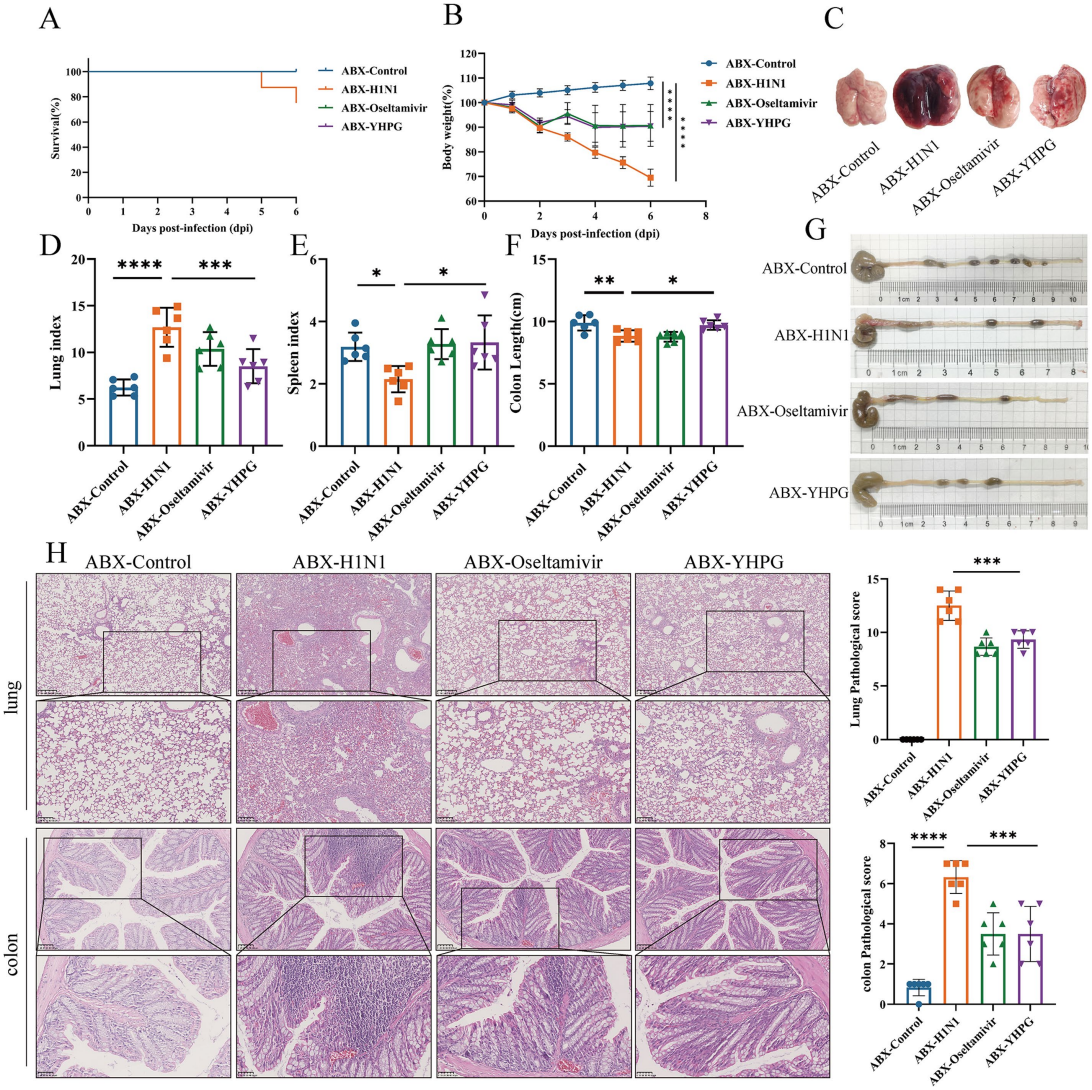
Effects of YHPG administration on IAV-infected mice with intestinal microbiota dysbiosis. **(A)** Survival rates of animals after infection. **(B)** Relative changes in body weight of mice during the experimental period. **(C)** Representative images of the overall morphology of mouse lung tissue (*n* = 6). **(D)** Lung index = Lung Weight (mg) / Body Weight (g). **(E)** Spleen index = Spleen Weight (mg) / Body Weight (g). **(F)** Statistics on colon length in mice. **(G)** Representative images of mouse colon length (*n* = 6). **(H)** Representative images of H&E staining in lung and intestine samples and the Pathological score (scale bars: 250 μm, 100 μm, 50 μm). *p < 0.05, **p < 0.01, ***p < 0.001, ****p < 0.0001.

To investigate the protective effect of YHPG on IAV-infected mice with dysbiota, gavage of a four-antibiotic (ABX) mixture prior to H1N1 infection was carried out in parallel with the previously mentioned experiment, resulting in intestinal microbiota depletion and dysbiosis. The ABX model was confirmed via anaerobic culture of intestinal contents and 16S sequencing (Supplementary Figures S1, S2). Following H1N1 infection in ABX mice, a more severe infection response was observed, with more pronounced pulmonary edema and congestion (Figure 2C). However, both the ABX-Oseltamivir and ABX-YHPG groups showed reduced weight loss, decreased lung index, increased spleen index, and increased colon length, with no significant difference between the two groups (Figures 2A–G; Supplementary Figure S4). Lung H&E staining revealed extensive neutrophil infiltration and inflammatory infiltration around small bronchi and blood vessels, along with stromal vascular dilation and widened alveolar septa. Treatment with YHPG and Oseltamivir significantly reduced inflammatory infiltration and vascular dilation (Figure 2H). In the colon, H&E staining indicated significant neutrophil infiltration, goblet cell necrosis, and capillary congestion in the H1N1 group. YHPG and Oseltamivir treatments decreased the inflammatory infiltration area (Figure 2H), suggesting that YHPG exhibits therapeutic effects in ABX IAV-infected mice, even in the context of intestinal microbiota dysbiosis.

### 3.3 YHPG Granules decrease viral load and inflammatory levels in ABX IAV-infected mice

**Figure 3:**
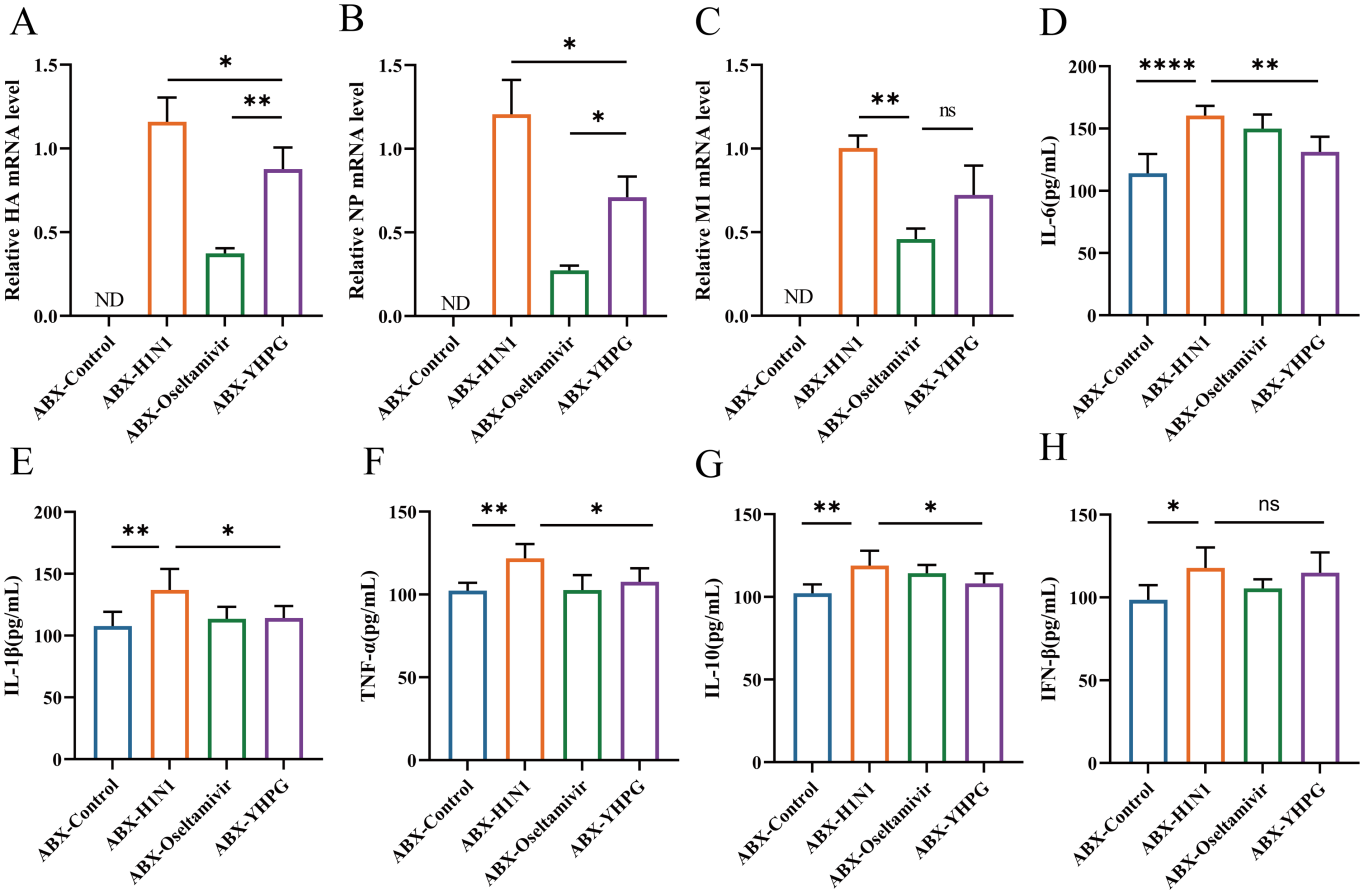
Effects of YHPG administration on viral load and inflammation levels in IAV-infected mice with intestinal microbiota dysbiosis. **(A)** mRNA expression levels of HA. **(B)** mRNA expression levels of NP. **(C)** mRNA expression levels of M1. **(D)** Levels of IL-6. **(E)** Levels of IL-1β. **(F)** Levels of TNF-α. **(G)** Levels of IL-10. **(H)** Levels of IFN-β. “ND” indicates not detectable, in HA, NP, M1 detection, ABX-H1N1 was normalized. *p < 0.05, **p < 0.01, ***p < 0.001, ****p < 0.0001.

RT-qPCR analysis showed that no H1N1-associated genes (HA, NP, or M1) were detected in the ABX-Control group, while viral titers were significantly lower in the YHPG and Oseltamivir groups compared to the ABX-H1N1 group (Figures 3A–C; Supplementary Figure S5). In addition, the levels of inflammatory cytokines in serum were assessed (Figures 3D–H). The ABX-H1N1 group exhibited significantly elevated serum levels of IL-10, TNF-α, IFN-β, IL-1β, and IL-6, while YHPG treatment led to a downward trend in these inflammatory markers. Notably, IFN-β levels remained elevated post-treatment. It has been reported that during intestinal inflammation, pro-inflammatory cytokines such as IL-1β and TNF-α are produced, affecting the expression of intestinal tight junction proteins, thereby altering intestinal barrier permeability and exacerbating intestinal inflammation (Kaminsky et al., 2021). This may contribute to a systemic inflammatory response. Following YHPG treatment, systemic inflammation markers such as IL-1β and TNF-α decreased, indicating that YHPG may modulate inflammatory factor levels, thereby alleviating colonic inflammation and barrier damage.

### 3.4 YHPG Granules improve colonic mucosal barrier damage in ABX IAV-infected mice

To verify the protective effect of YHPG on the colonic mucosal barrier in mice, we observed the expression of tight junction proteins in the colon via immunofluorescence staining.

**Figure 4:**
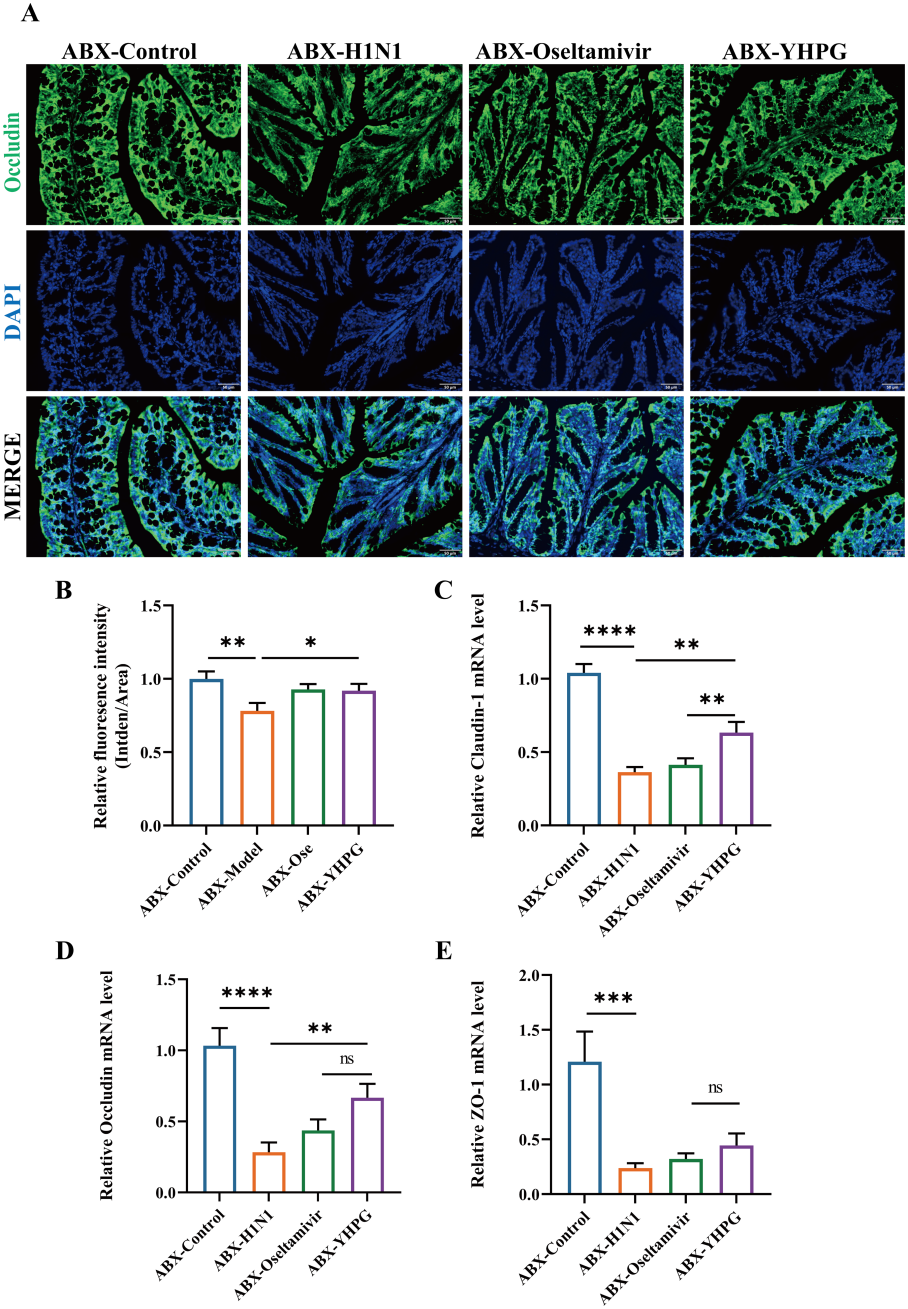
The effect of YHPG administration on intestinal barrier dysfunction in IAV-infected mice with dysregulated gut microbiota. **(A)** Immunofluorescence staining results of Occludin (green), DAPI (blue), and merged images in the colon, scale bar: 50 μm. **(B)** Semi-quantitative results of Occludin in the colon. **(C)** mRNA expression levels of Claudin-1. **(D)** mRNA expression levels of Occludin. **(E)** mRNA expression levels of ZO-1. *p < 0.05, **p < 0.01, ***p < 0.001, ****p < 0.0001.

The results indicated that compared to the control group, the expression of the tight junction protein Occludin in the intestinal epithelial cells of the ABX-H1N1 group was irregular and relatively decreased. In contrast, YHPG treatment increased the relative expression of Occludin (Figures 4A,B). Moreover, mRNA expression levels of the tight junction proteins Occludin, Claudin-1, and ZO-1 were elevated in the intestines of ABX IAV mice treated with YHPG compared to the ABX-H1N1 group (Figures 4C–E). These findings suggest that YHPG Granules have the potential to improve intestinal barrier dysfunction.

### 3.5 YHPG Granules modulate the gut microbiota composition and increase microbial diversity in ABX IAV mice

**Figure 5:**
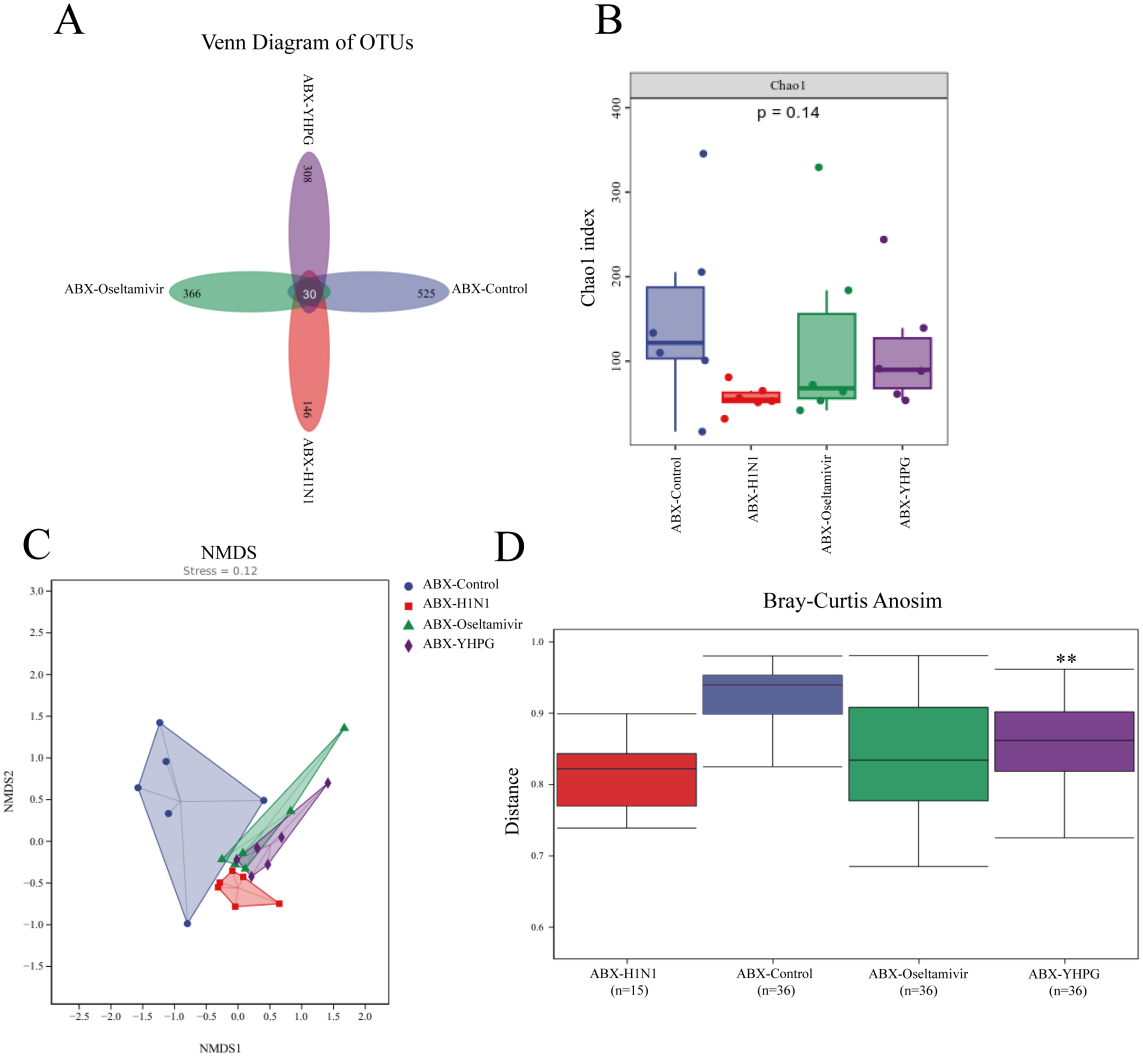
The effect of YHPG administration on the gut microbiota composition in ABX IAV mice. **(A)** Venn diagram based on ASVs, with numbers indicating the values of OTUs detected in all mice within a group. **(B)** Chao1 index. **(C)** NMDS analysis. Stress = 0.12 < 0.2 (it is generally believed that when the value is less than 0.2, the results of NMDS analysis are more reliable). **(D)** Analysis of inter-group differences. *R* = 0.246296, **p < 0.01 compared to ABX-H1N1.

To explore the relationship between the colonic mucosal barrier and gut microbiota, we performed 16S rRNA gene sequencing on fecal samples from all four ABX-treated groups. A comparative analysis of the resulting data revealed significant differences in gut microbiota structure between the ABX-H1N1 and ABX-YHPG groups. Specifically, 525 ASVs were detected in the ABX-Control group, 146 ASVs in the ABX-H1N1 group, and 308 ASVs in the ABX-YHPG group (Figure 5A), indicating a decrease in species diversity in ABX mice post-infection, while YHPG helped restore microbiota diversity to some extent. The Chao1 index showed decreased biodiversity in infected ABX mice, and although the Chao1 index of ABX-YHPG did not differ significantly from the model group, it exhibited an upward trend, suggesting a recovery potential with YHPG treatment (Figure 5B). NMDS analysis indicated that the microbial community structure in ABX-YHPG mice differed more from that of ABX-H1N1 mice than from ABX-Oseltamivir mice, suggesting that YHPG treatment led to greater differentiation in microbial communities (Figure 5C). Inter-group analysis confirmed that the distance between the ABX-YHPG and ABX-H1N1 groups was greater than that between the ABX-Oseltamivir and ABX-H1N1 groups (Figure 5D).

### 3.6 YHPG Granules increase the abundance of beneficial bacteria in ABX IAV mice, particularly those producing SCFAs

**Figure 6:**
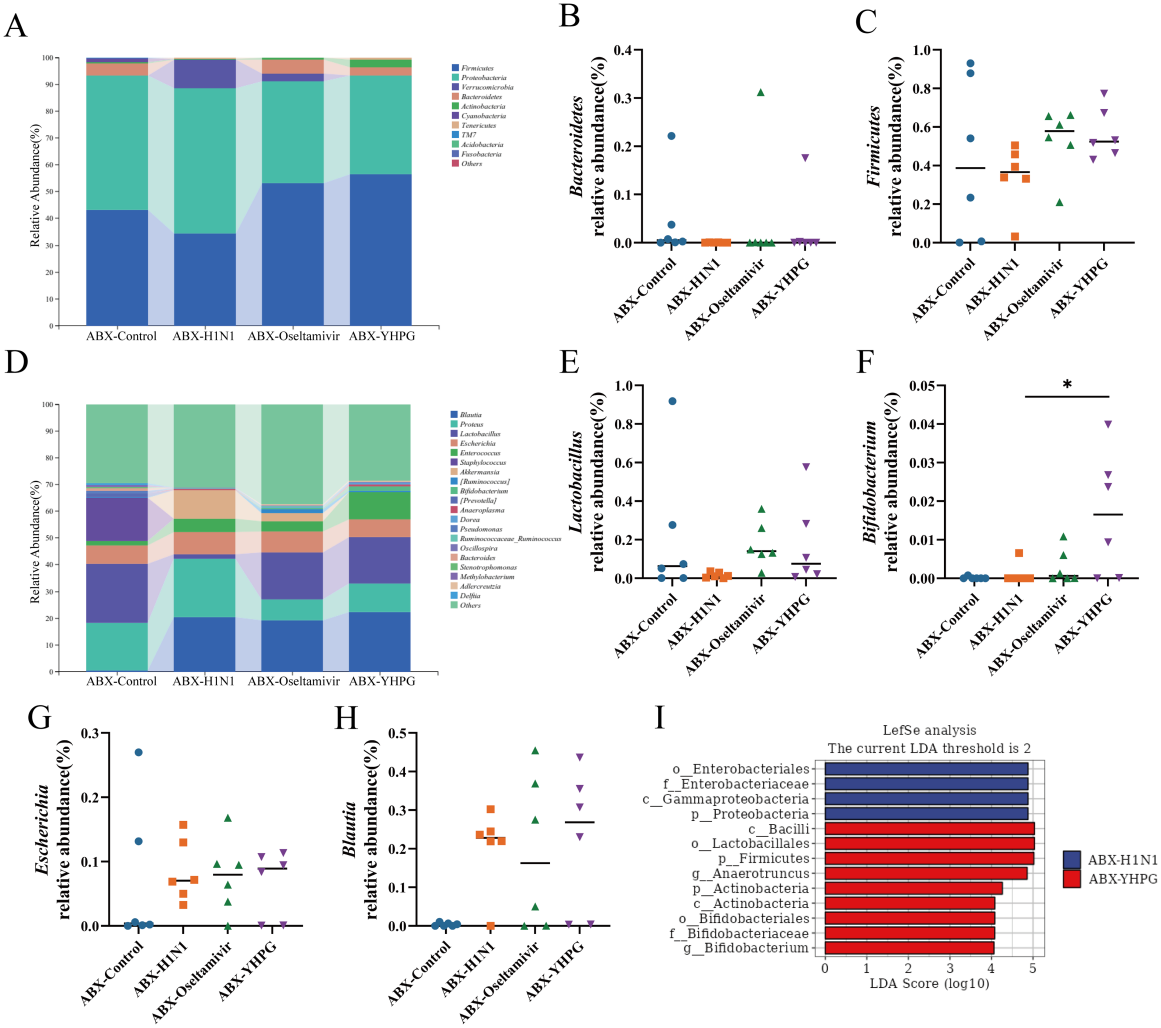
The effect of YHPG administration on gut microbiota at different taxonomic levels in ABX IAV mice. **(A)** Relative abundance of species at the phylum level. **(B)** Relative abundance of *Bacteroidetes*. **(C)** Relative abundance of *Firmicutes*. **(D)** Relative abundance at the genus level. **(E)** Relative abundance of *Lactobacillus*. **(F)** Relative abundance of *Bifidobacterium*. **(G)** Relative abundance of *Escherichia*. **(H)** Relative abundance of *Blautia*. **(I)** LefSe analysis between the YHPG treatment group and the model group. Data were presented as mean ± standard deviation and Kruskal-Wallis test was performed by Dunnett’s method. Compared with ABX-H1N1 group, *p < 0.05.

At the phylum level, *Firmicutes* and *Bacteroidetes* were the predominant phyla in all tested samples, with no statistically significant difference in their relative abundances (Figures 6A–C). At the genus level, YHPG treatment significantly increased the relative abundance of *Bifidobacterium*, along with other short-chain fatty acid (SCFA)-producing genera such as *Blautia*, *Dorea*, and *Oscillospira*, indicating that YHPG may influence SCFA levels in mice. Additionally, the relative abundance of pathogenic genera such as *Escherichia* decreased following YHPG treatment (Figures 6D–H). LEfSe analysis identified specific genera between the ABX-YHPG and ABX-H1N1 groups, with the ABX-YHPG group showing enrichment in *Lactobacillus*, *Bifidobacterium*, *Actinobacteria*, and *Bacilli* (Figure 6I).

### 3.7 YHPG Granules alter the metabolic characteristics in ABX IAV mice, with acetate and propionate identified key SCFAs

**Figure 7:**
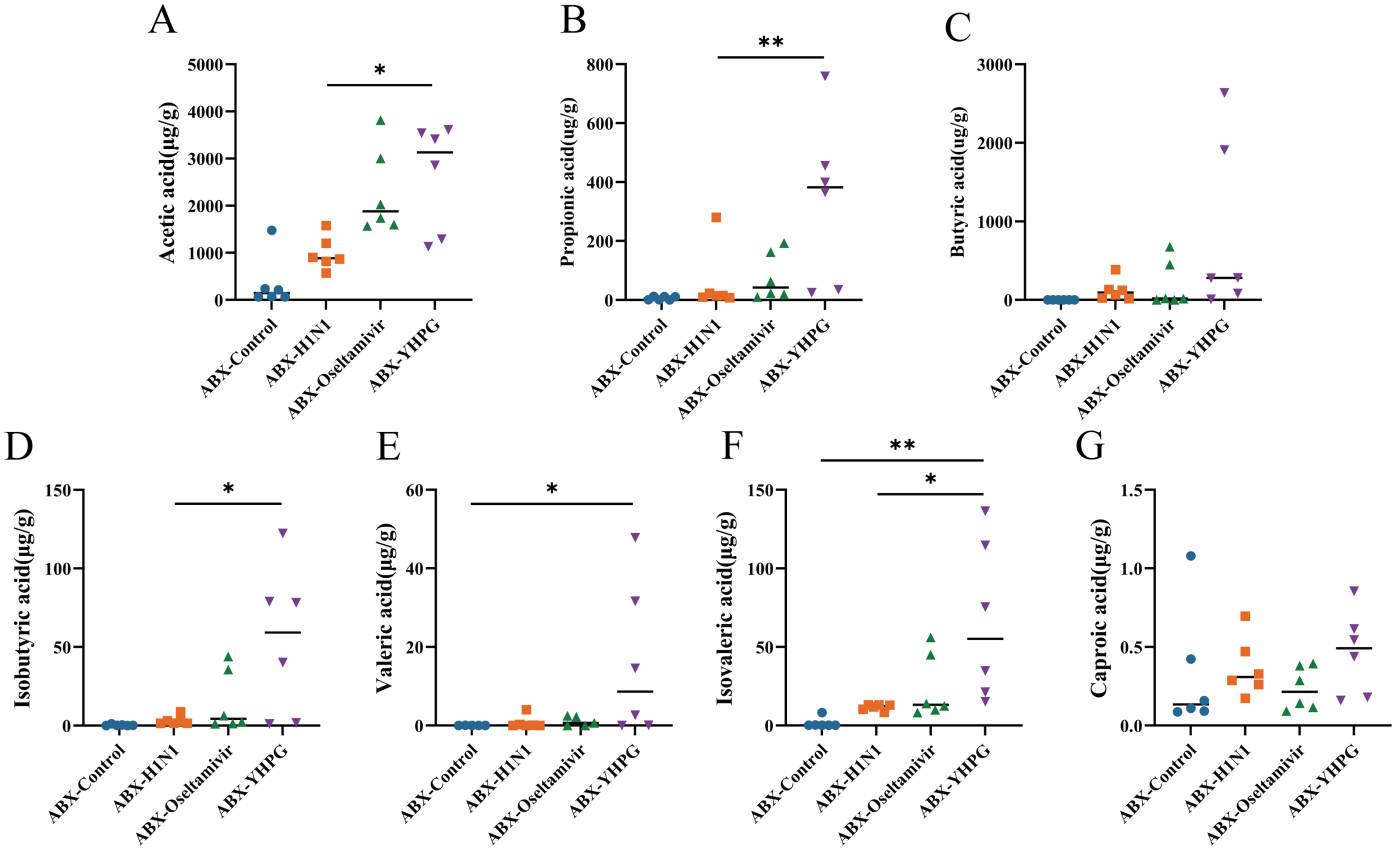
The effect of YHPG on the SCFA content in the feces of ABX IAV mice. **(A)** Acetic acid level. **(B)** Propionic acid level. **(C)** Isobutyric acid level. **(D)** Butyric acid level. **(E)** Isovaleric acid level. **(F)** Valeric acid level. **(G)** Caproic acid level. Data were presented as mean ± standard deviation and Kruskal-Wallis test was performed by Dunnett’s method. Compared with ABX-H1N1 group, *p < 0.05, **p < 0.01.

To determine whether the changes in gut microbiota affect SCFA metabolism in mice, we conducted targeted metabolomics to detect seven common SCFAs in the colon. Comparing the metabolic profiles of ABX IAV mice and YHPG-treated mice, we found that all SCFA levels were elevated in the ABX-YHPG group, with significant increases in acetate, propionate, isobutyrate, and isovalerate (Figures 7A–G). In contrast, SCFA levels in the ABX-Oseltamivir group did not show a significant difference or upward trend compared to the ABX-H1N1 group, suggesting that Oseltamivir did not improve fecal SCFA levels.

### 3.8 Correlation analysis of gut microbiota, gut microbiome-related metabolomics, and influenza indicators identifies potential biomarkers of YHPG treatment for H1N1 influenza virus

**Figure 8:**
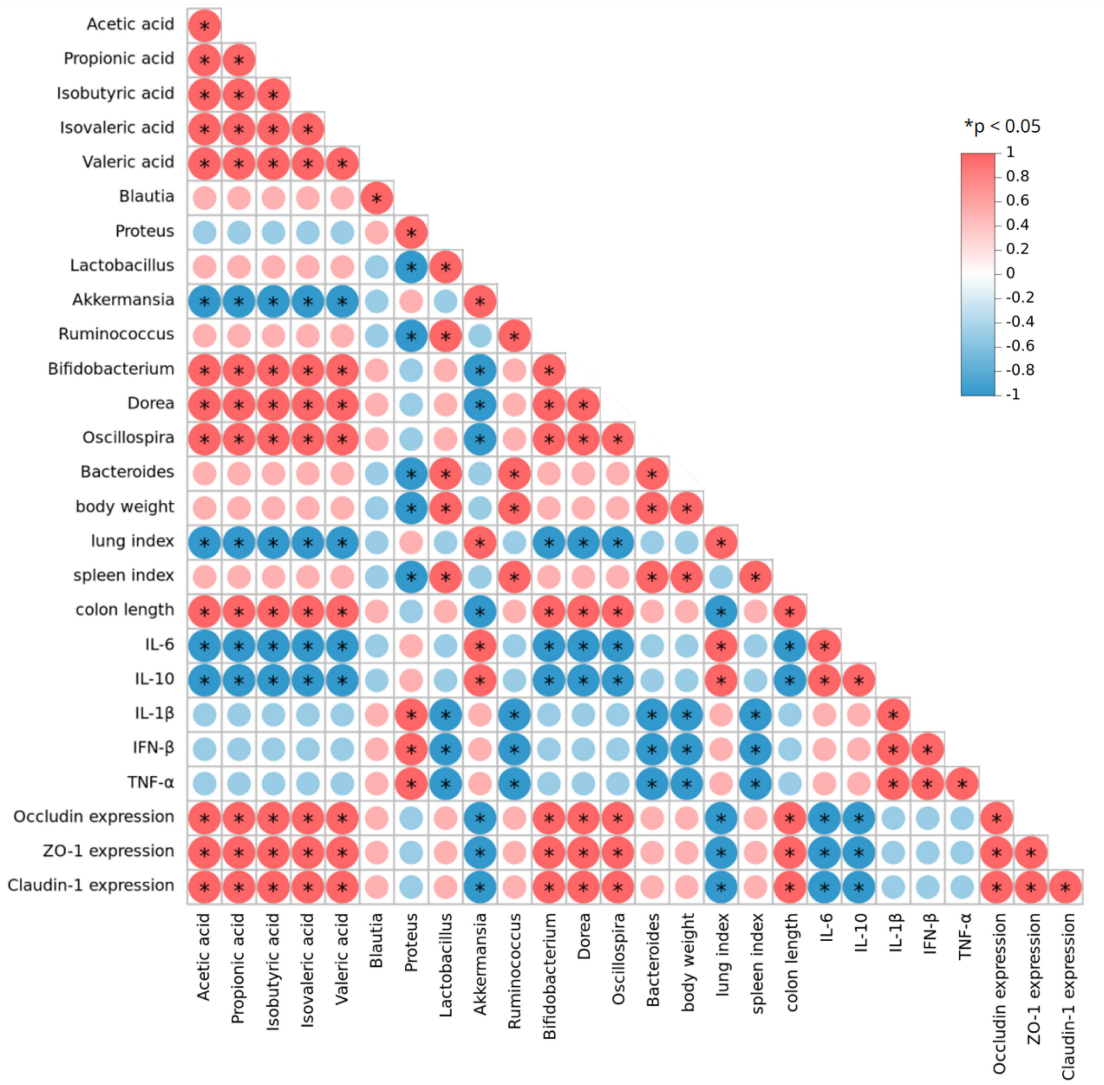
Spearman correlation analysis of gut microbiota, SCFAs, and indicators related to H1N1 infection. Red indicates a positive correlation, while blue indicates a negative correlation. *p < 0.05.

To comprehensively explore the relationship between conventional influenza-related indicators and gut microbiota, we performed a Spearman correlation analysis on experimental data, focusing on gut microbiota composition, SCFA levels, and infection-related indicators after YHPG treatment (Figure 8). The analysis revealed positive correlations between certain intestinal microbiota and influenza-related indices. For instance, *Bifidobacterium*, Dorea, *Oscillospira*, *Lactobacillus* and *Bacteroides* were positively correlated with the intestinal tight junction protein Occludin, while both genera showed a negative correlation with Inflammatory cytokines. Additionally, the SCFAs acetate and propionate were positively associated with the expression levels of *Bifidobacterium*, Dorea and *Oscillospira*.

### 3.9 YHPG Granules activate the GPR43-MAVS-IRF3-IFN-I pathway by modulating gut microbiota metabolites, specifically SCFAs

SCFAs have been reported to mediate the oligomerization of MAVS by binding to G protein-coupled receptors, thereby facilitating the phosphorylation that activates the TBK1/IRF3 pathway. Previous research highlighted the overexpression of IFN-β following YHPG treatment. To further investigate the effect of YHPG on the GPR43-MAVS-IRF3-IFN-I pathway, we measured the expression of pathway-related proteins in the colon using RT-qPCR and Western blot.

**Figure 9:**
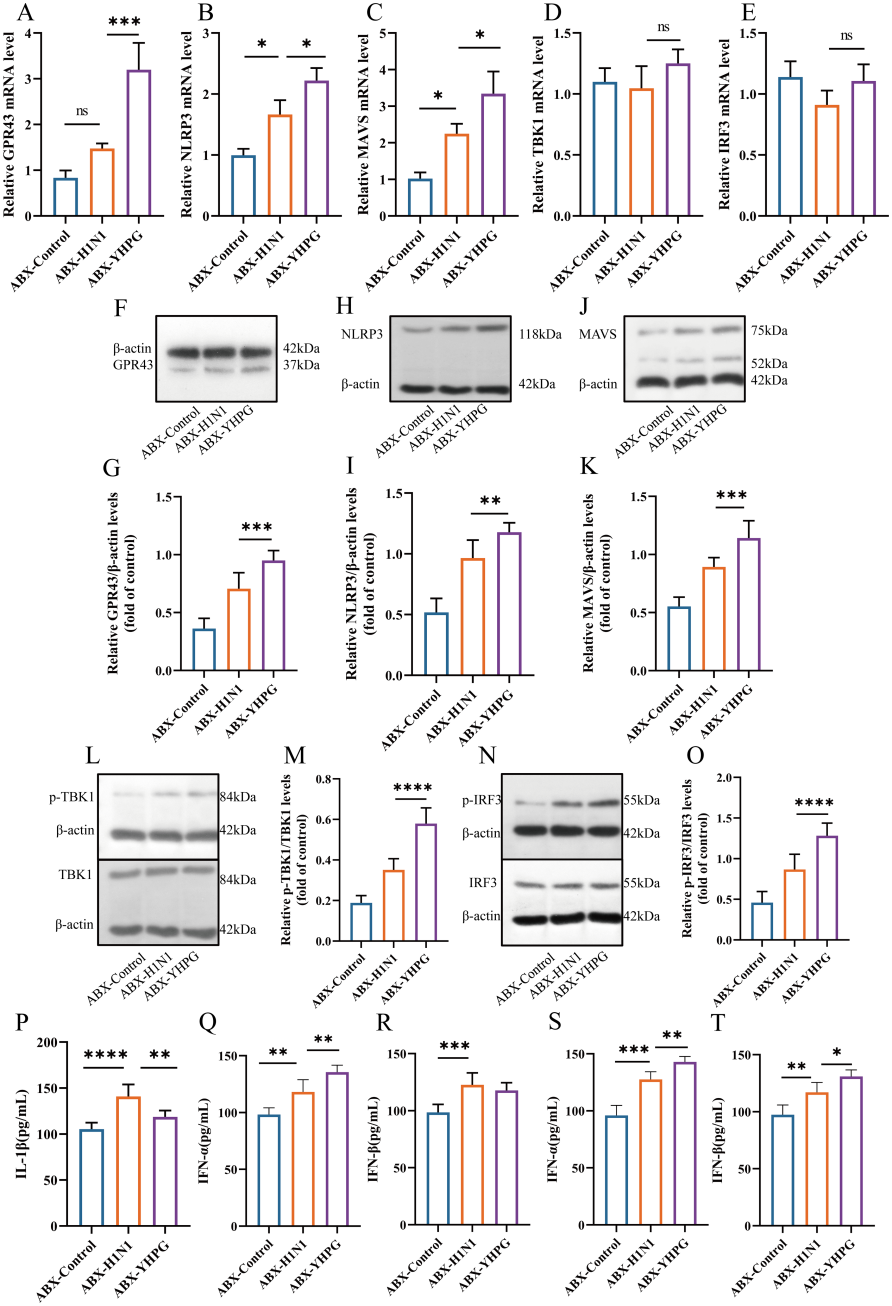
YHPG may enhance the antiviral response of the host through the GPR43-MAVS-IRF3-IFN-I pathway. **(A)** mRNA expression levels of GPR43. **(B)** mRNA expression levels of NLRP3. **(C)** mRNA expression levels of MAVS. **(D)** mRNA expression levels of TBK1. **(E)** mRNA expression levels of IRF3. **(F)** Representative Western blot images of GPR43. **(G)** Relative expression ratio of GPR43 to β-actin. **(H)** Representative Western blot images of NLRP3. **(I)** Relative expression ratio of NLRP3 to β-actin. **(J)** Representative Western blot images of MAVS. **(K)** Relative expression ratio of MAVS to β-actin. **(L)** Representative Western blot images of p-TBK1 and TBK1. **(M)** Relative expression ratio of p-TBK1 to TBK1. **(N)** Representative Western blot images of p-IRF3 and IRF3. **(O)** Relative expression ratio of p-IRF3 to IRF3. **(P)** Levels of IL-1β in the colon. **(Q)** Levels of IFN-α in the colon. **(R)** Levels of IFN-β in the colon. **(S)** Levels of IFN-α in the lung. **(T)** Levels of IFN-β in the lung. *p < 0.05, **p < 0.01, ***p < 0.001, ****p < 0.0001.

As shown in Figures 9A–E, Supplementary Figure S6, the expression levels of GPR43, NLRP3, MAVS, TBK1, and IRF3 proteins were higher in the ABX-H1N1 group compared to the ABX-Control group. This may be caused by inflammatory stimulation after viral infection. However, YHPG treatment significantly increased the expression of genes associated with this pathway. Western blot analysis confirmed these findings (Figures 9F–K), where the ratios of p-TBK1/TBK1 and p-IRF3/IRF3 following YHPG treatment showed significant differences compared to the ABX-H1N1 group (Figures 9L–O). This suggests that YHPG may activate the GPR43-MAVS-IRF3-IFN-I pathway by modulating SCFAs, leading to TBK1 and IRF3 phosphorylation. Additionally, it has been reported that phosphorylated IRF3 transcribes IFN-β in the nucleus, which is subsequently released (Ko et al., 2022). The increased levels of IL-1β and type I interferons in both intestinal and lung tissues indirectly confirm that YHPG may activate the IRF3 pathway (Figures 9P–T).

## 4 Discussion

The gut microbiota is considered an important regulator of human health, and recent studies have shown that gut microbiota plays an increasingly important role in combating influenza A virus infections (Harper et al., 2020; Ou et al., 2023). The balance and diversity of gut microbiota not only affect the host’s immune response but also enhance antiviral capacity by regulating metabolites such as short-chain fatty acid. Therefore, maintaining and regulating the health of the gut microbiota may become a new strategy for preventing and treating IAV infections. This research direction not only introduces new pathways for antiviral treatments but also offers new insights into integrating traditional Chinese medicine with modern scientific research.

In this study, antibiotic administration exacerbated host pathology during influenza infection, likely due to reduced levels of pro-inflammatory immune responses. YHPG treatment alleviated pulmonary and intestinal pathological conditions, increased the expression of intestinal tight junction proteins, and reduced the release of pro-inflammatory factors, confirming its therapeutic efficacy against influenza under conditions of gut microbiota dysbiosis. After YHPG administration, the Chao1 index showed an upward trend, while the relative abundance of pathogenic bacteria (*Escherichia*, *Enterobacteriaceae*) decreased, and SCFA-producing bacteria (*Bacteroides*, *Akkermansia*, *Bifidobacterium*, *Lactobacillus*) significantly increased. Targeted metabolomics further confirmed elevated SCFA levels, including acetate and propionate.

*Lactobacilli*, as key gut probiotics, can enhance interferon signaling capacity, and their derived fatty acids enhance the host’s ability to resist pathogens (Kim et al., 2023). *Bifidobacterium*-derived SCFAs have immune-activating effects, promoting dendritic cell IL-10 secretion, enhancing immune cell activity, and inducing a balance in Th1/Th2 immune responses (Mahooti et al., 2019; Zhang et al., 2020). *Dorea* and *Blautia* participate in carbohydrate fermentation, producing SCFAs and maintaining normal barrier function (Becker et al., 2011). *Oscillospira*, widely present in animal and human intestines, produces butyrate and other SCFAs and is considered a next-generation probiotic candidate. *Actinobacteria,* with protective effects against influenza and pneumonia, hold potential as probiotics for preventing severe secondary pneumonia following influenza infection (Wang et al., 2024). These findings suggest that YHPG may exert its anti-influenza effects by modulating the abundance of probiotics and increasing host SCFA levels.

Gut probiotics produce SCFAs, such as acetate, propionate, and butyrate, which stimulate the growth of probiotics by regulating the environmental pH, forming a positive feedback loop (Liu et al., 2019). SCFAs also serve as critical energy sources for intestinal epithelial cells, playing anti-inflammatory and immunoregulatory roles in the gut (Sivaprakasam et al., 2016; Hills et al., 2019). Acetates and propionates can induce neutrophil chemotaxis through the activation of the cell surface receptor GPR43 (Sun et al., 2017), while acetate can cause MAVS aggregation via GPR43, leading to downstream TBK1 and IRF3 phosphorylation (Niu et al., 2023). MAVS receives upstream signals from PRRs in the RNA-sensing pathway, inducing IFN production through prion-like aggregates. Activation and phosphorylation of IRF3 initiate type I interferon expression, such as IFN-β (Tang and Wang, 2009), which strongly inhibits influenza virus propagation. Our RT-qPCR and Western blot analyses showed that YHPG can upregulate GPR43, NLRP3, and MAVS expression, leading to TBK1 and IRF3 phosphorylation.

In conclusion, these findings demonstrate that gut microbiota significantly influence lung index, spleen index, colon length, weight changes, and inflammatory factor levels in hosts post-infection. YHPG may protect the intestinal barrier and reduce host inflammation by adjusting gut microbiota composition and SCFA levels, contributing to the restoration of a healthier gut microenvironment and mitigating influenza progression.

However, this study has some limitations. The study was conducted in ABX mice, and despite strict controls for the mice’s diet and other factors, the gut microbiota can be affected clinically by many uncontrollable factors. Therefore, the results of this study cannot be directly applied to the clinic, and the model has certain limitations in fully representing the population with clinical intestinal dysfunction. In the selection of animal models, the subsequent use of GPR43 knockout mice could provide a more direct approach to investigate the relationship between SCFAs and GPR43 signaling. In future studies, integrating metagenomic sequencing, bacterial *in vitro* cultures, and fecal microbiota transplantation may help further analyze the regulatory effects of YHPG on gut microbiota, along with associated SCFA production and the expression of key functional genes.

## 5 Conclusion

**Figure 10:**
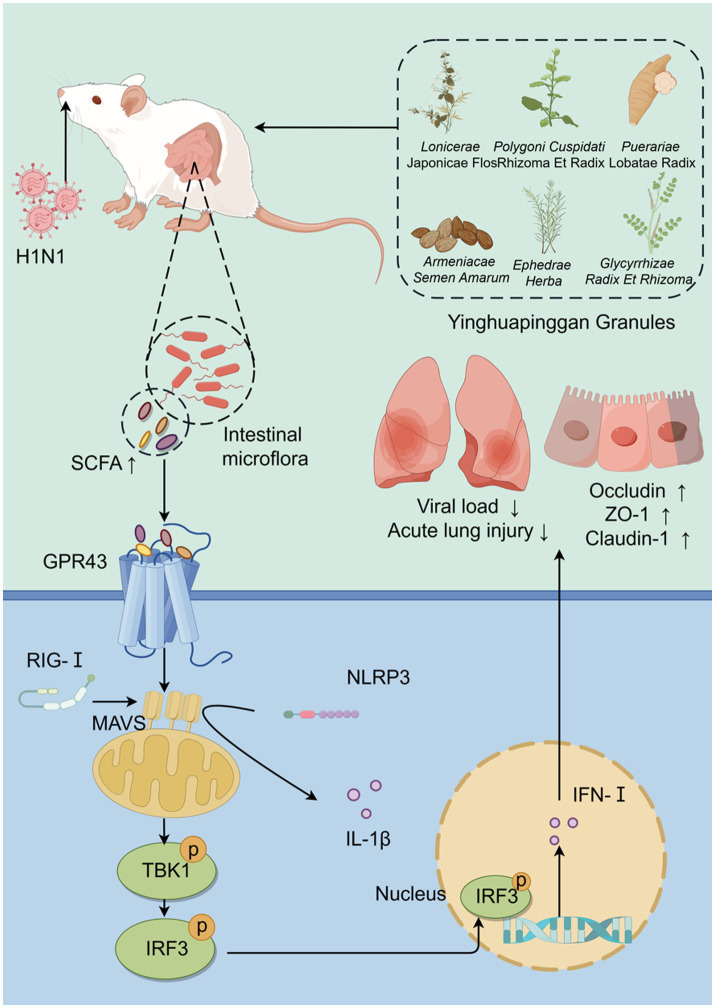
Yinhua Pinggan Granules alleviate lung and intestinal damage in influenza virus-infected mice by modulating gut microbiota and its metabolites to activate the GPR43-MAVS-IRF3-IFN-β pathway.

In summary (Figure 10), this study demonstrates that YHPG enhances the antiviral capacity of IAV-infected mice with disrupted gut microbiota. YHPG alleviates acute lung injury, protects the colonic barrier, modulates gut microbiota composition, and alters SCFA levels in mice. Pharmacologically, YHPG may activate the GPR43-MAVS-IRF3-IFN-β pathway by increasing SCFA levels, thereby promoting host recovery.

## Data Availability

The datasets presented in this study can be found in online repositories. The names of the repository/repositories and accession number(s) can be found in the article/Supplementary material.

## References

[ref1] Antunes K. H. Fachi J. L. de Paula R. da Silva E. F. Pral L. P. dos Santos A. Á.. (2019). Microbiota-derived acetate protects against respiratory syncytial virus infection through a GPR43-type 1 interferon response. Nat. Commun. 10:3273. doi: 10.1038/s41467-019-11152-6, PMID: 31332169 PMC6646332

[ref2] Becker N. Kunath J. Loh G. Blaut M. (2011). Human intestinal microbiota: characterization of a simplified and stable gnotobiotic rat model. Gut Microbes 2, 25–33. doi: 10.4161/gmic.2.1.14651, PMID: 21637015

[ref3] Creager H. M. Kumar A. Zeng H. Maines T. R. Tumpey T. M. Belser J. A. (2018). Infection and replication of influenza virus at the ocular surface. J. Virol. 92:e02192-17. doi: 10.1128/jvi.02192-17, PMID: 29321303 PMC5972870

[ref4] Dai J.-P. Wang Q.-W. Su Y. Gu L.-M. Zhao Y. Chen X.-X.. (2017). Emodin inhibition of influenza A virus replication and influenza viral pneumonia via the Nrf2, TLR4, p38/JNK and NF-kappaB pathways. Molecules 22:1754. doi: 10.3390/molecules2210175 29057806 PMC6151665

[ref5] Du H.-X. Zhou H.-F. Yang J.-H. Lu Y.-Y. He Y. Wan H.-T. (2020). Preliminary study of Yinhuapinggan granule against H1N1 influenza virus infection in mice through inhibition of apoptosis. Pharm. Biol. 58, 979–991. doi: 10.1080/13880209.2020.1818792, PMID: 32962483 PMC7534346

[ref6] Du H.-X. Zhu J.-Q. Chen J. Zhou H.-F. Yang J.-H. Wan H.-T. (2021). Revealing the therapeutic targets and molecular mechanisms of emodin-treated coronavirus disease 2019 via a systematic study of network pharmacology. Aging 13, 14571–14589. doi: 10.18632/aging.203098, PMID: 34088885 PMC8221358

[ref7] Feng C. Jin C. Liu K. Yang Z. (2023). Microbiota-derived short chain fatty acids: their role and mechanisms in viral infections. Biomed. Pharmacother. 160:114414. doi: 10.1016/j.biopha.2023.114414

[ref8] Gu Y. Hsu A. C.-Y. Pang Z. Pan H. Zuo X. Wang G.. (2019). Role of the innate cytokine storm induced by the Influenza A virus. Viral Immunol. 32, 244–251. doi: 10.1089/vim.2019.0032, PMID: 31188076

[ref9] Harper A. Vijayakumar V. Ouwehand A. C. Ter Haar J. Obis D. Espadaler J.. (2020). Viral Infections, the Microbiome, and Probiotics. Front. Cell. Infect. Microbiol. 10:596166. doi: 10.3389/fcimb.2020.596166, PMID: 33643929 PMC7907522

[ref10] He Y. Wan H. Zhou H. Yang J. Yu L. Li C.. (2019). Safety and effectiveness clinical research of randomized double blinded and positive drug of parallel control and multicenter on clearing away lung heat and dispersing lung qi formula to treat lung qi stagnation syndrome due to exogenous pathogenic heat. China J. Tradit. Chin. Med. Pharm. 34, 5972–5977. [in Chinese].

[ref11] Hills R. D. Pontefract B. A. Mishcon H. R. Black C. A. Sutton S. C. Theberge C. R. (2019). Gut microbiome: profound implications for diet and disease. Nutrients 11:1613. doi: 10.3390/nu11071613, PMID: 31315227 PMC6682904

[ref12] Iwasaki A. Pillai P. S. (2014). Innate immunity to influenza virus infection. Nat. Rev. Immunol. 14, 315–328. doi: 10.1038/nri3665, PMID: 24762827 PMC4104278

[ref13] Javanian M. Barary M. Ghebrehewet S. Koppolu V. Vasigala V. Ebrahimpour S. (2021). A brief review of influenza virus infection. J. Med. Virol. 93, 4638–4646. doi: 10.1002/jmv.26990, PMID: 33792930

[ref14] Kaminsky L. W. Al-Sadi R. Ma T. Y. (2021). IL-1β and the Intestinal Epithelial Tight Junction Barrier. Front. Immunol. 12:767456. doi: 10.3389/fimmu.2021.767456, PMID: 34759934 PMC8574155

[ref15] Karami A. Fakhri S. Kooshki L. Khan H. (2022). Polydatin: Pharmacological Mechanisms, Therapeutic Targets, Biological Activities, and Health Benefits. Molecules 27:6474. doi: 10.3390/molecules27196474, PMID: 36235012 PMC9572446

[ref16] Kim S. Lee S. Kim T.-Y. Lee S.-H. Seo S.-U. Kweon M.-N. (2023). Newly isolated *Lactobacillus paracasei* strain modulates lung immunity and improves the capacity to cope with influenza virus infection. Microbiome 11:260. doi: 10.1186/s40168-023-01687-8, PMID: 37996951 PMC10666316

[ref17] Ko R. Seo J. Park H. Lee N. Lee S. Y. (2022). Pim1 promotes IFN-β production by interacting with IRF3. Exp. Mol. Med. 54, 2092–2103. doi: 10.1038/s12276-022-00893-y, PMID: 36446848 PMC9722908

[ref18] Langer D. Mlynarczyk D. T. Dlugaszewska J. Tykarska E. (2023). Potential of glycyrrhizic and glycyrrhetinic acids against influenza type A and B viruses: A perspective to develop new anti-influenza compounds and drug delivery systems. Eur. J. Med. Chem. 246:114934. doi: 10.1016/j.ejmech.2022.114934, PMID: 36455358

[ref19] Liu X. Wu C. Han D. Liu J. Liu H. Jiang Z. (2019). Partially Hydrolyzed Guar Gum Attenuates d-Galactose-Induced Oxidative Stress and Restores Gut Microbiota in Rats. Int. J. Mol. Sci. 20:4861. doi: 10.3390/ijms20194861, PMID: 31574948 PMC6801633

[ref20] Mahooti M. Abdolalipour E. Salehzadeh A. Mohebbi S. R. Gorji A. Ghaemi A. (2019). Immunomodulatory and prophylactic effects of *Bifidobacterium bifidum* probiotic strain on influenza infection in mice. World J. Microbiol. Biotechnol. 35:91. doi: 10.1007/s11274-019-2667-0, PMID: 31161259

[ref9001] Ni Z. Wang J. Yu X. Wang Y. Wang J. He X.. (2024). Influenza virus uses mGluR2 as an endocytic receptor to enter cells. Nat Microbiol. 9, 1764–1777. doi: 10.1038/s41564-024-01713-x 38849624 PMC11222159

[ref21] Niu J. Cui M. Yang X. Li J. Yao Y. Guo Q.. (2023). Microbiota-derived acetate enhances host antiviral response via NLRP3. Nat. Commun. 14:642. doi: 10.1038/s41467-023-36323-4, PMID: 36746963 PMC9901394

[ref22] Ou G. Xu H. Wu J. Wang S. Chen Y. Deng L.. (2023). The gut-lung axis in influenza A: the role of gut microbiota in immune balance. Front. Immunol. 14:1147724. doi: 10.3389/fimmu.2023.1147724, PMID: 37928517 PMC10623161

[ref23] Peng X.-Q. Zhou H.-F. Lu Y.-Y. Chen J.-K. Wan H.-T. Zhang Y.-Y. (2016b). Protective effects of Yinhuapinggan granule on mice with influenza viral pneumonia. Int. Immunopharmacol. 30, 85–93. doi: 10.1016/j.intimp.2015.11.029, PMID: 26655878

[ref24] Peng X. Zhou H. Zhang Y. Yang J. Wan H. He Y. (2016a). Antiviral effects of Yinhuapinggan granule against influenza virus infection in the ICR mice model. J. Nat. Med. 70, 75–88. doi: 10.1007/s11418-015-0939-z, PMID: 26439479

[ref25] Singh A. K. Singla R. K. Pandey A. K. (2023). Chlorogenic Acid: A Dietary Phenolic Acid with Promising Pharmacotherapeutic Potential. Curr. Med. Chem. 30, 3905–3926. doi: 10.2174/0929867329666220816154634, PMID: 35975861

[ref26] Sivaprakasam S. Prasad P. D. Singh N. (2016). Benefits of short-chain fatty acids and their receptors in inflammation and carcinogenesis. Pharmacol. Ther. 164, 144–151. doi: 10.1016/j.pharmthera.2016.04.007, PMID: 27113407 PMC4942363

[ref27] Smith P. M. Howitt M. R. Panikov N. Michaud M. Gallini C. A. Bohlooly-Y M.. (2013). The microbial metabolites, short-chain fatty acids, regulate colonic Treg cell homeostasis. Science 341, 569–573. doi: 10.1126/science.1241165, PMID: 23828891 PMC3807819

[ref28] Steed A. L. Christophi G. P. Kaiko G. E. Sun L. Goodwin V. M. Jain U.. (2017). The microbial metabolite desaminotyrosine protects from influenza through type I interferon. Science 357, 498–502. doi: 10.1126/science.aam5336, PMID: 28774928 PMC5753406

[ref29] Stefan K. L. Kim M. V. Iwasaki A. Kasper D. L. (2020). Commensal Microbiota Modulation of Natural Resistance to Virus Infection. Cell 183, 1312–1324.e10. doi: 10.1016/j.cell.2020.10.047, PMID: 33212011 PMC7799371

[ref30] Subramanian N. Natarajan K. Clatworthy M. R. Wang Z. Germain R. N. (2013). The Adaptor MAVS Promotes NLRP3 Mitochondrial Localization and Inflammasome Activation. Cell 153, 348–361. doi: 10.1016/j.cell.2013.02.054, PMID: 23582325 PMC3632354

[ref31] Sun M. Wu W. Liu Z. Cong Y. (2017). Microbiota metabolite short chain fatty acids, GPCR, and inflammatory bowel diseases. J. Gastroenterol. 52, 1–8. doi: 10.1007/s00535-016-1242-9, PMID: 27448578 PMC5215992

[ref32] Swanson K. V. Deng M. Ting J. P.-Y. (2019). The NLRP3 inflammasome: molecular activation and regulation to therapeutics. Nat. Rev. Immunol. 19, 477–489. doi: 10.1038/s41577-019-0165-0, PMID: 31036962 PMC7807242

[ref33] Tang E. D. Wang C.-Y. (2009). MAVS Self-Association Mediates Antiviral Innate Immune Signaling. J. Virol. 83, 3420–3428. doi: 10.1128/jvi.02623-08, PMID: 19193783 PMC2663242

[ref34] te Velthuis A. J. W. Fodor E. (2016). Influenza virus RNA polymerase: insights into the mechanisms of viral RNA synthesis. Nat. Rev. Microbiol. 14, 479–493. doi: 10.1038/nrmicro.2016.87, PMID: 27396566 PMC4966622

[ref35] Wang L. Chen M. Sun Q. Yang Y. Rong R. (2023). Discovery of the potential neuraminidase inhibitors from *Polygonum cuspidatum* by ultrafiltration combined with mass spectrometry guided by molecular docking. J. Sep. Sci. 46:e2200937. doi: 10.1002/jssc.202200937, PMID: 36905353

[ref36] Wang G. Liu J. Zhang Y. Xie J. Chen S. Shi Y.. (2023). Ginsenoside Rg3 enriches SCFA-producing commensal bacteria to confer protection against enteric viral infection via the cGAS-STING-type I IFN axis. ISME J. 17, 2426–2440. doi: 10.1038/s41396-023-01541-7, PMID: 37950067 PMC10689736

[ref37] Wang S. Ou G. Wu J. Chen Y. Xu L. Xu H. (2024). Genetically Predicted Peripheral Immune Cells Mediate the Effect of Gut Microbiota on Influenza Susceptibility. Int. J. Mol. Sci. 25:7706. doi: 10.3390/ijms25147706, PMID: 39062949 PMC11276963

[ref38] Wang H.-X. Zeng M.-S. Ye Y. Liu J.-Y. Xu P.-P. (2021). Antiviral activity of puerarin as potent inhibitor of influenza virus neuraminidase. Phytother. Res. 35, 324–336. doi: 10.1002/ptr.6803, PMID: 32757226

[ref39] Yalkun I. Wan H. Ye L. Yu L. He Y. Li C.. (2024). Qualitative and Quantitative Analysis of Chemical Components in Yinhua Pinggan Granule with High-Performance Liquid Chromatography Coupled with Q-Exactive Mass Spectrometry. Molecules 29:2300. doi: 10.3390/molecules29102300, PMID: 38792164 PMC11124461

[ref40] Yang C. Chen J. Zhou H. Zeng D. Wan H. Yang J. (2024). Therapeutic effect of Yinhuapinggan granules mediated through the intestinal flora in mice infected with the H1N1 influenza virus. Front. Microbiol. 15:1394304. doi: 10.3389/fmicb.2024.1394304, PMID: 38741735 PMC11089240

[ref41] Zhang Q. Hu J. Feng J.-W. Hu X.-T. Wang T. Gong W.-X.. (2020). Influenza infection elicits an expansion of gut population of endogenous *Bifidobacterium animalis* which protects mice against infection. Genome Biol. 21:99. doi: 10.1186/s13059-020-02007-1, PMID: 32345342 PMC7187530

[ref42] Zhu Z. Fodor E. Keown J. R. (2023). A structural understanding of influenza virus genome replication. Trends Microbiol. 31, 308–319. doi: 10.1016/j.tim.2022.09.01 36336541

