# Supplementary material for: Yinhua Pinggan Granules alleviate lung and intestinal damage in influenza virus-infected mice by modulating gut microbiota and its metabolites to activate the GPR43-MAVS-IRF3-IFN-β pathway
Source: Front Microbiol. 2025 Oct 6;16:1532108. doi: 10.3389/fmicb.2025.1532108 (PMC12535982; doi:10.3389/fmicb.2025.1532108)
Supplement: Supplementary file 1 [file Supplementary_file_1.docx]

Supplementary Material

# Supplementary Figures and Tables


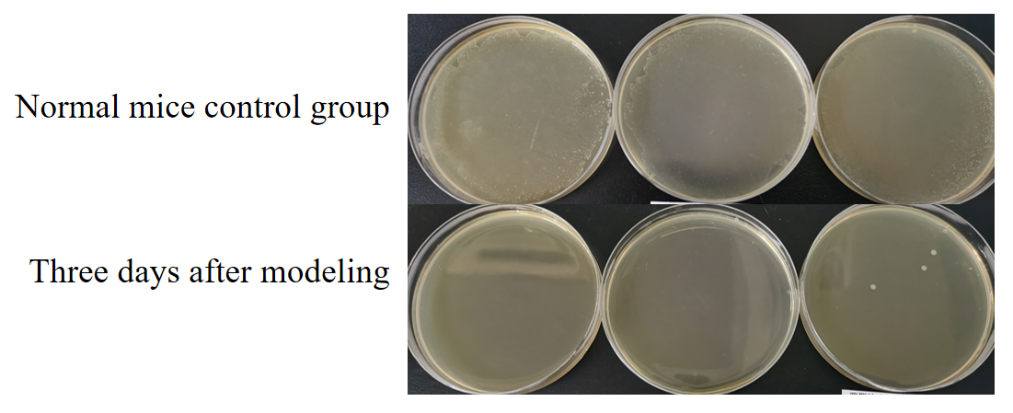


**Supplementary Figure 1.** After modeling, the feces of ABX mice were taken to prepare 50 mg / ml suspension for anaerobic culture.


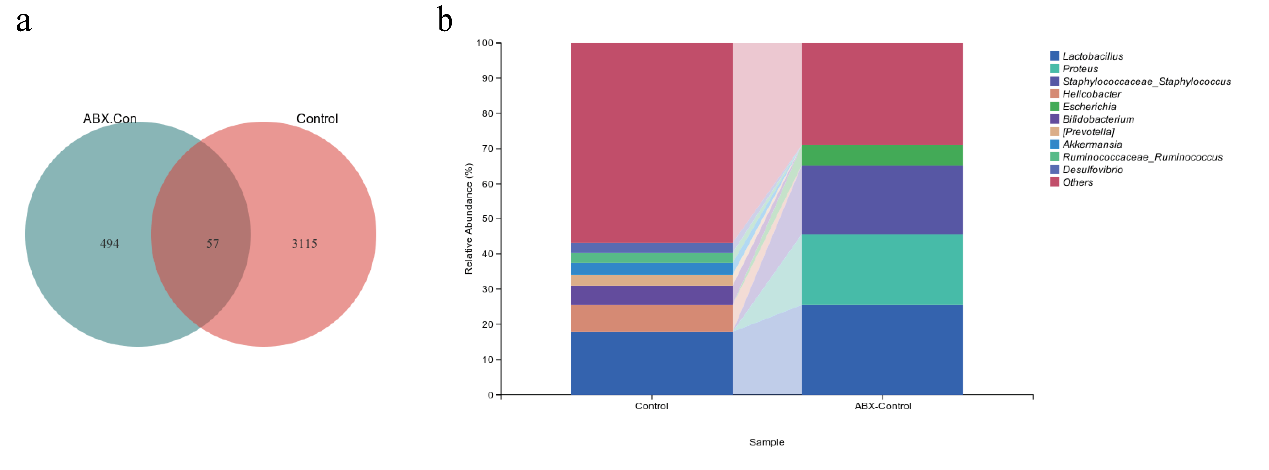


**Supplementary Figure 2.** The feces of ABX mice were taken for 16S sequencing after modeling. (a) Venn diagram. (b) Relative abundance at the genus level.


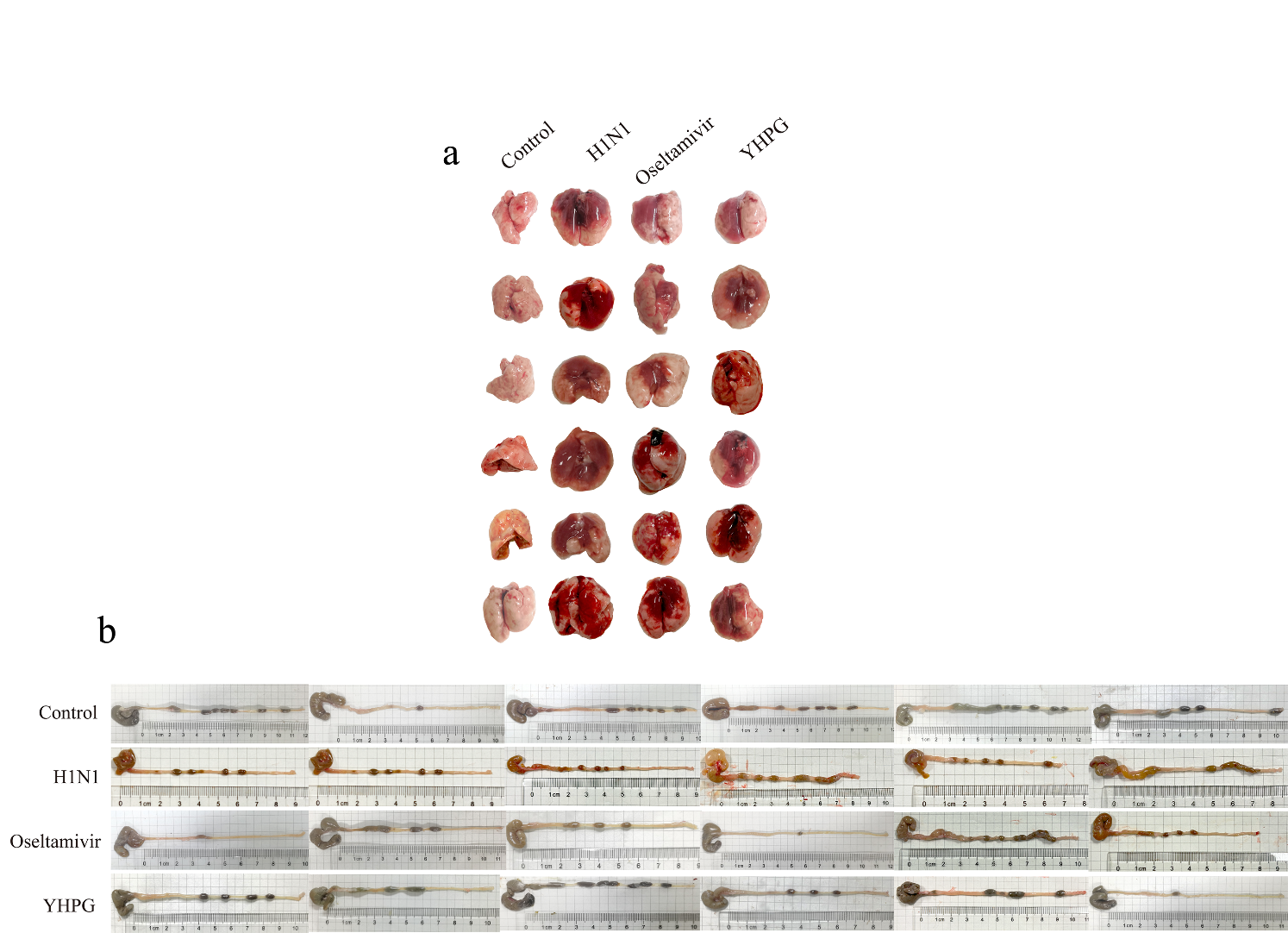


**Supplementary Figure 3.** Lung and intestinal tissues from IAV-infected mice. (a) Images of the overall morphology of mouse lung tissue. (b) Images of mouse colon length.


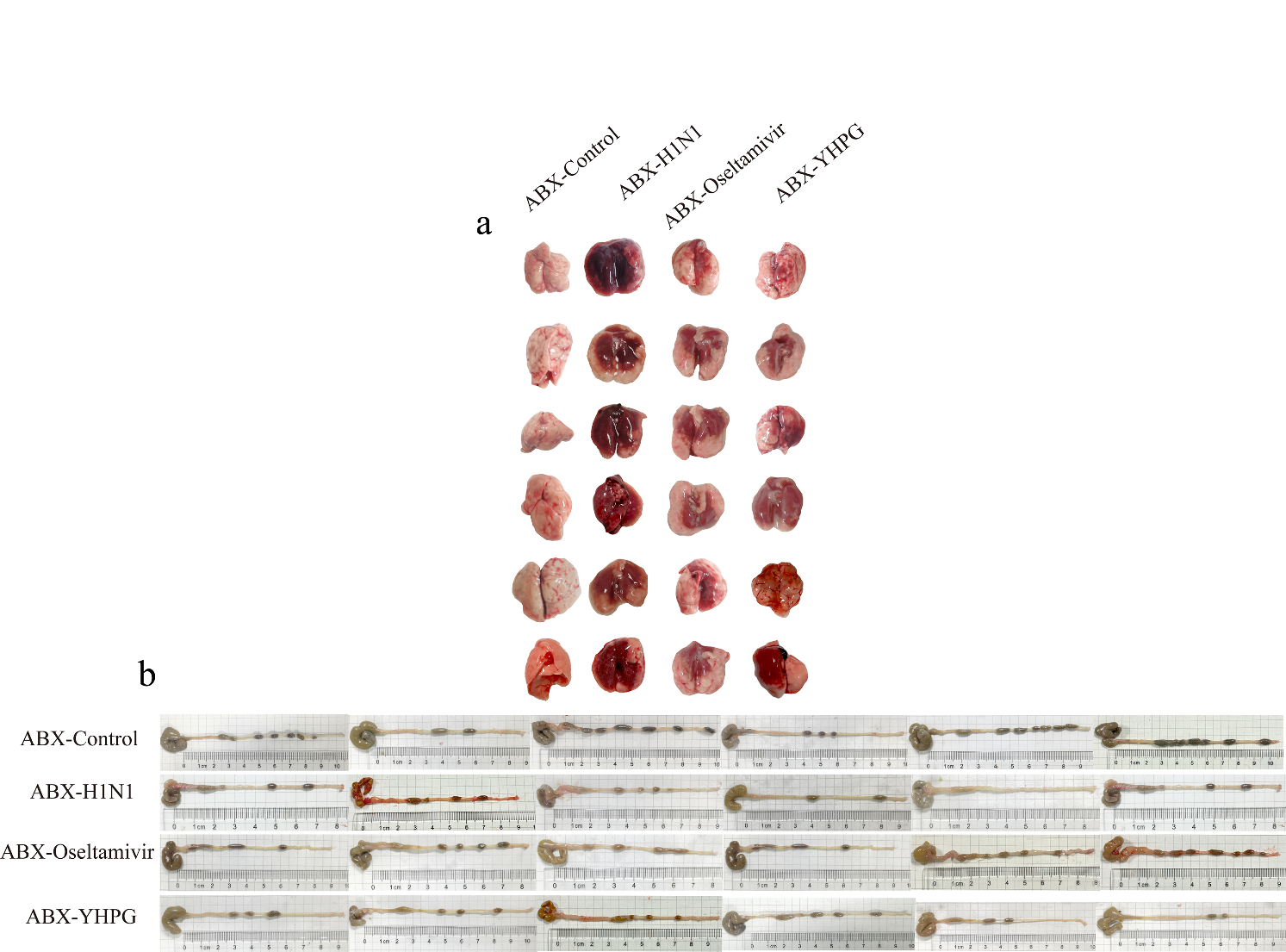


**Supplementary Figure 4.** Lung and intestinal tissues from IAV-infected ABX mice. (a) Images of the overall morphology of mouse lung tissue. (b) Images of mouse colon length.


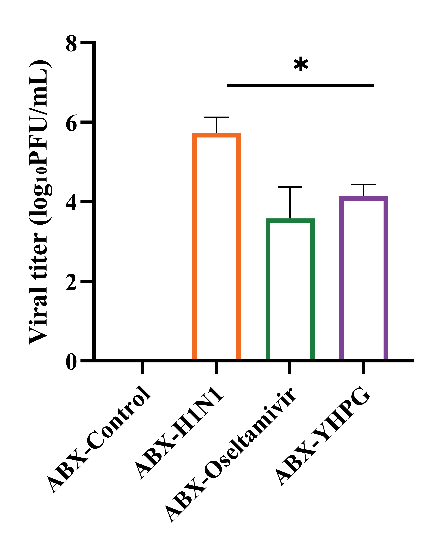


**Supplementary Figure 5**. The lung titration.


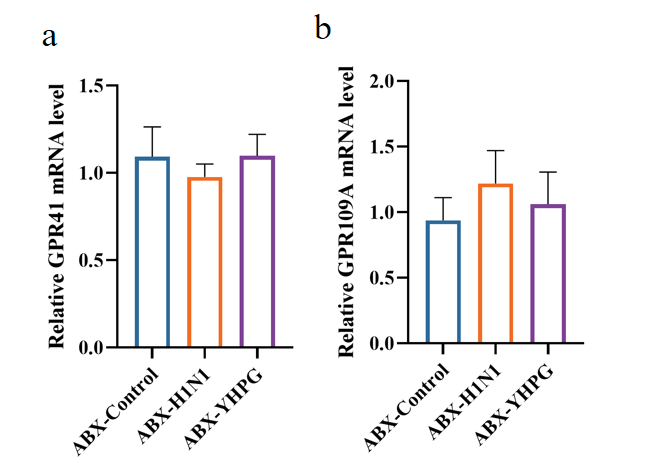


**Supplementary Figure 6.** (a) mRNA expression levels of GPR41. (b) mRNA expression levels of GPR109A.
